# Cytological Findings of Cylindroma: Differential Diagnosis and Literature Review

**DOI:** 10.7759/cureus.74648

**Published:** 2024-11-28

**Authors:** Junpei Wada, Haruto Nishida, Tsutomu Daa, Kenji Kashima, Aiko Kato, Shogo Urabe

**Affiliations:** 1 Pathology, Oita Prefectural Hospital, Oita, JPN; 2 Diagnostic Pathology, Faculty of Medicine, Oita University, Oita, JPN; 3 Laboratory Medicine, Oita Prefectural Hospital, Oita, JPN; 4 Plastic and Reconstructive Surgery, Oita Prefectural Hospital, Oita, JPN

**Keywords:** dermal cylindroma, fine-needle aspiration, nuclear atypia, skin neoplasm, sputum cytology

## Abstract

A cylindroma is a relatively rare tumor classified as a benign tumor with apocrine and eccrine differentiation, mainly occurring on the scalp. While there are some reports on the cytological findings of cylindromas, there are no reports concerning stamp preparation. Here, we report a case of a 60-year-old female who presented with a tumor on the right scalp. We obtained cytological specimens from non-fixed surgical samples using stamp, aspiration, and liquid-based cytology. Based on the histological findings, we compared the similarities and differences between stamp and aspiration preparations of a typical cylindroma. Findings of basement membrane-like materials and the degree of nuclear atypia aided cytological diagnosis. As distinguishing cylindromas from other tumors through cytology alone is clinically challenging, careful diagnosis using histopathology and cytology is recommended.

## Introduction

Typical skin tumors of the head and neck region include sebaceous nevi, poromas, spiradenomas, pleomorphic adenomas, basal cell adenomas, basal cell carcinomas, and adenoid cystic carcinomas; however, cylindromas are rare. First reported in 1842 by Ancell, a cylindroma is a benign skin tumor arising from the sweat gland. It commonly occurs in the head and neck region, especially the scalp [1]. While there are some reports on cylindroma cytological findings using the aspiration method, there are no reports on using the stamp preparation method [2-10]. Furthermore, distinguishing cylindromas from other tumors is challenging because of their rarity. Here, we report the cytological findings of a case of cylindroma using stamp, aspiration, and liquid-based cytology specimens and provide a literature review to discuss the differences between the two approaches.

## Case presentation

Case history/examination

A 60-year-old female presented with a tumor on the right side of the scalp that had been present for five years. The tumor was slow-growing and slightly painful. The patient visited our hospital for further evaluation. She had undergone surgery to remove a serous carcinoma of the endometrium two years prior and had no recurrence. Macroscopic examination revealed a well-defined 8 mm × 8 mm, tender, and solitary nodular tumor on the right side of the scalp. A biopsy and subsequent histopathological analysis led to a cylindroma diagnosis. The tumor was subsequently excised.

Cytological and histopathological investigations

**Figure 1:**
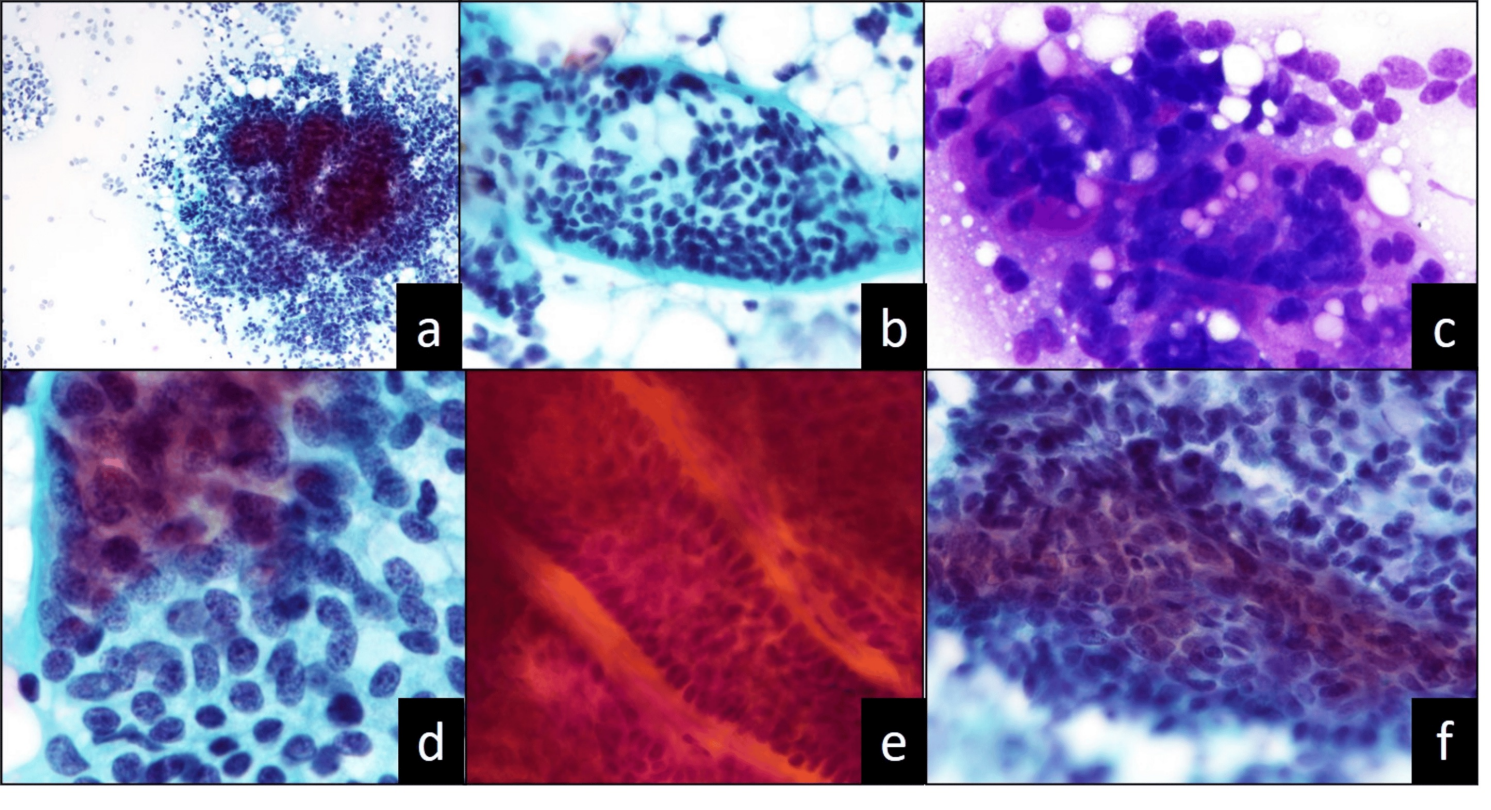
Stamp (a–d) and aspiration preparations (e–f) of cylindroma specimens: (a, b, d–f) Papanicolaou stain; (c) May–Giemsa stain (a) The clear background and high level of cellularity (x10), (b) basement membrane-like material and palisading arrangement in the periphery of the cluster (x20), (c) metachromasia in the basement membrane-like or globular materials (x40), (d) two types of cells (dark and small or bright and large) in the cluster (x40), (e) tubular structure (x20), and (f) streaming feature (x40).

We obtained cytological specimens from non-fixed surgical samples using stamp, aspiration, and liquid-based cytology (LBC; SurePath, Beckton Dickinson, Franklin Lakes, NJ). The stamp specimen exhibited high cellularity, but its background was clear (Figure 1, Panel a). Large-to-small irregularly shaped clusters were found. The tumor cells partially spilled from the border of the clusters. At the periphery of the clusters, the tumor cells presented a palisading arrangement, and basement membrane-like materials surrounded the clusters (Figure 1, Panel b). Globular materials were also observed inside the clusters. The materials were periodic acid-Schiff-positive, and May-Giemsa staining revealed metachromasia (Figure 1, Panel c). The tumor cells had scant cytoplasm and smooth round-to-oval nuclei with fine chromatin; however, the nucleoli were inconspicuous. Dark and bright nuclei were observed. The dark nuclei were slightly smaller than the bright ones present at the periphery of the clusters and exhibited a palisade arrangement. The bright nuclei were located inside the cluster (Figure 1, Panel d). The aspiration and LBC specimens showed findings similar to those of the stamp specimens. However, a cluster with a tubular structure or streaming feature was observed in the aspiration specimen (Figure 1, Panels e and f). May-Giemsa staining revealed metachromasia in the materials in these clusters.

**Figure 2:**
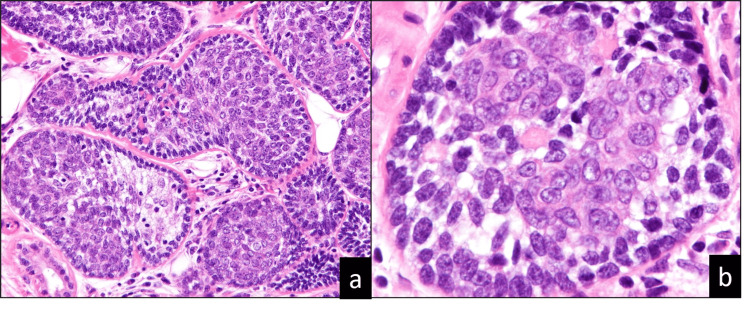
The histopathological findings The tumor cells form clusters with a jigsaw puzzle-like appearance. (a) Basement membrane-like materials are around and inside the clusters of tumor cells and are divided into different compartments (x20). (b) The tumor cells are small, round to oval, with hyperchromatic nuclei, inconspicuous nucleoli, and scant cytoplasm (x40).

Macroscopically, the tumor exhibited a tan-gray and multinodular pattern on the cut surface. Histopathologically, the tumor was well-circumscribed and showed high cellularity, with multiple nodules from the dermis to the subcutaneous fat tissue. The tumor cells formed clusters with a jigsaw puzzle-like appearance. Basement membrane-like materials were found around and inside the clusters of tumor cells, which were divided into different compartments (Figure 2, Panel a). The tumor cells were small, round to oval, with hyperchromatic nuclei, inconspicuous nucleoli, and scant cytoplasm (Figure 2, Panel b). The clusters exhibited a palisading arrangement of nuclei and two types of cells. No nuclear atypia, increased mitotic activity (including atypical mitosis), necrosis, or invasion was observed.

Diagnosis and clinical outcome

Based on the cytological and histological findings, the tumor was diagnosed as a cylindroma. Although the tumor margin was positive, the tumor did not recur for one year.

## Discussion

Cylindromas occur commonly in the head and neck region, especially the scalp, and rarely in the trunk, extremities, or external genital regions. This distribution pattern corresponds to that of the apocrine glands. Cylindromas predominantly affects middle-aged females [9]. The tumor can be painful at times, and multiple cylindromas can occur simultaneously on the scalp. In familial cases, cylindromas are associated with other skin adnexal tumors, such as spiradenoma and trichoepithelioma (Brooke-Spiegler syndrome). CYLD on chromosome 16q has been implicated in Brooke-Spiegler syndrome. Spiradenomas and cylindromas are related types of the same tumor. Since cylindromas and spiradenomas commonly show genetic abnormalities in CYLD and histological transitions, these tumors are also collectively referred to as spiradenocylindroma.

Skin tumors are easily biopsied, but their cytology has rarely been reported [2-10]. Some studies have reported the cytological findings of cylindromas, which are summarized in Table 1.

**Table 1:** Differential diagnosis of cylindroma CYLD: Cylindroma; ACC: Adenoid cystic carcinoma; SPA: Spiradenoma; BCC: Basal cell carcinoma; BM: Basement membrane.

	Chromatin	Degree of nuclear atypia	Cluster of two cells	Palisading	BM-like materials	Background
CYLD	Dense-sparse, fine	Low	Present	Present	Surrounding or spherical	Clear
ACC	Dense-sparse	Low-high	Present	Absent	Variety of colors and forms	Mucous balls (necrosis)
SPA	Sparse	Low	Absent	Absent	Spherical	Cylindromatous component
BCC	Dense, coarse	High	Absent	Present	Not present	Amyloid, melanin, and necrosis

Many cell clusters with clear or bloody backgrounds were observed in these studies. Cells were cohesive and formed sheet-like, tubular, or streaming clusters. The tumor cells were small and had a high nuclear/cytoplasmic ratio. Nuclei were smoothly round or oval and had fine, dark (thick), or bright (thin) chromatin. Dark cells showed a palisading arrangement at the periphery, and bright cells were located at the center of the clusters. Nucleoli were inconspicuous. No nuclear membrane thickening or atypical mitosis was observed. Basement-membrane-like materials were also observed. The materials were membranous around the clusters, and small spheres were present at the center. They were periodic acid-Schiff-positive and displayed metachromasia as revealed by May-Giemsa or toluidine blue staining [4,9]. These findings were also observed in the present case.

The aforementioned findings are based on aspiration preparations. To the best of our knowledge, no reports have been published on stamp preparation for cylindromas. This may be because aspiration cytology and biopsies are often conducted during preoperative diagnosis. However, a tumor may be discovered, for instance, during intraoperative diagnosis.

Herein, stamp and aspiration preparations produced remarkably similar results. Hence, it is important to report the cytological findings of stamp preparations. Compared with aspiration cytology, stamp cytology preserves cellular morphology but exhibits some differences. In detail, we were able to evaluate the findings such as the background surrounding clusters, the cellularity of the clusters, nuclear findings (membrane thickening, nuclear swelling, irregularity, chromatin pattern, and nucleoli), lack of atypical mitosis, and basement membrane-like materials, but the tubular or streaming figure was not observed in the stamp in our case. These differences are likely due to variations in tissue sampling or the frequency of findings rather than the preparation method itself.

Differential diagnosis of cylindromas is important for determining a treatment strategy. Aspiration and LBC are useful because they can be performed easily and are minimally invasive. The main differential diagnoses are cutaneous adnexal tumors, such as primary cutaneous adenoid cystic carcinoma (pcACC) and spiradenoma [7,8]. These tumors exhibit cell patterns, such as cell size, nuclear shape, nucleus/cytoplasm ratio, and nucleoli, similar to those of cylindromas at low magnification [7,8]. In particular, tumor cells of ACC show hyperchromatic nuclei, but in other tumors, the nuclei show hypochromic or mixed patterns, as was observed in our case. Cribriform structures are characteristic of pcACCs, and basement membrane-like materials exhibit a variety of colors and forms in pcACCs. Mucous balls are detected in pcACCs but not in other tumors. They form small spheric clusters with no surroundings, as is the case in spiradenoma. However, the basement membrane-like structures are surrounding/inside the clusters in cylindromas. Basal cell carcinomas may need to be distinguished from cylindromas, which differ in nuclear atypia, amyloid, and, occasionally, melanin deposits (Table 1) [11]. As these tumors resemble each other and cylindroma-like components can emerge, especially in spiradenomas, it may be very difficult to distinguish these tumors using cytology alone. Therefore, it is important to consider the limitations of a cytological diagnosis and, thus, refer to histopathological findings.

Malignant cylindromas should be distinguished from benign cylindromas. To date, little has been reported about the cytology of malignant cylindromas. However, malignant cells generally show coarser chromatin and higher nuclear atypia than benign cells. In the present case, as there was no cytological atypia, the patient was diagnosed with a benign cylindroma.

## Conclusions

We report a rare case of cylindroma that was investigated based on the cytological findings of stamp and aspiration preparations. The emerging characteristics of the basement membrane-like materials surrounding/inside the clusters aided the differential diagnosis. The appearance of materials can be clinching features for diagnosis. It is clinically challenging to distinguish cylindromas from other tumors based on cytology alone. Therefore, they should be carefully diagnosed, taking histopathology into consideration.
